# Effect of Size-Distribution Environment on Breakage Parameters Using Closed-Cycle Grinding Tests

**DOI:** 10.3390/ma16247687

**Published:** 2023-12-17

**Authors:** Evangelos Petrakis

**Affiliations:** School of Mineral Resources Engineering, University Campus, Technical University of Crete, Kounoupidiana, 73100 Chania, Greece; evpetrakis@tuc.gr; Tel.: +30-28210-37608

**Keywords:** grinding kinetics, breakage parameters, closed-cycle circuit, energy efficiency, particle size distribution, simulation

## Abstract

The so-called population balance model (PBM) is the most widely used approach to describe the grinding process. The analysis of the grinding data is carried out using—among others—the one-size fraction BII method. According to the BII method, the breakage parameters can be determined when a narrow particle size fraction is used as feed material to the mill. However, it is commonly accepted that these parameters are influenced by changing the particle size distribution in the mill. Thus, this study examines the breakage parameters through kinetic testing in different natural-size distribution environments generated by closed-cycle grinding tests that simulate industrial milling conditions. The differentiation of the milling environments was accomplished using various reference sieves in the closed-cycle tests. The experimentally determined breakage parameters were back-calculated and then used to simulate the closed-cycle tests using the MODSIM^TM^ software. Additionally, the energy efficiency was evaluated based on the specific surface area of the grinding products and the energy consumption. The results of the kinetic tests showed that the breakage rate of the coarse particles increases as the aperture size of the reference sieve decreases, and consequently, the content of fines in the mill increases. The back-calculated breakage parameters can be reliably used to simulate closed-cycle circuits, thus helping control industrial milling operations.

## 1. Introduction

Comminution is the process of size reduction widely used in many industrial sectors, including mineral processing. Its main purpose is to produce the desired size and liberate the minerals of interest from other invaluable constituents so that they can be efficiently separated [1]. However, comminution, especially grinding, is the most energy-intensive process in mining operations, accounting for more than 50% of the total energy used [2,3,4]. Also, grinding is characterized by low efficiency and high carbon emissions. This has led mining companies to set targets to be more environmentally friendly and significantly reduce their operational carbon footprint, typically by up to 30–40% over the next 10–15 years [5]. These targets will require mining companies to develop new alternative technologies and operate by promoting green and climate-smart practices [6,7].

In grinding, for instance, many efforts have been made over the past 150 years to improve the efficiency of the process. The mathematical models proposed in the scientific literature start with the historical empirical energy-size reduction relationships (referred to as laws of comminution) of Rittinger, Kick and Bond [8], while in recent years, much attention has been paid to the machine learning techniques, including artificial neural networks (ANNs) [9]. ANNs are inspired by the brain structure and consist of several elements called neurons. In addition, kinetic models derived from population balance considerations have garnered a significant amount of interest. The so-called population balance model (PBM), which uses two functions, namely the breakage rate (or selection function) *S*_i_ and breakage function *b*_i,j_, provides the fundamental size–mass balance equation for fully mixed batch grinding operations [10,11,12]. Its advantages, including process control and scale-up of laboratory tests to industrial mills, have been highlighted by numerous studies [13,14,15,16]. The PBM considers that the breakage rate *S*_i_ of size class *i* is time-invariant, and grinding follows a first-order law expressed as [17,18],

(1) $$\frac{{dm}_{i}(t)}{dt}=-S_{i}\cdot m_{i}(t)$$

or

(2) $$m_{i}(t)=m_{i}(0)\cdot exp(-S_{i}\cdot t)$$

where *m*_i_(t) and *m*_i_(0) are the mass fractions for size class *i* at grinding time *t* and *0*, respectively.

According to Austin et al. [12], the breakage rate *S*_i_ (min^−1^) can be described by a power function of particle size, as shown in Equation (3),

(3) $$S_{i}=\alpha_{T}\cdot x^{\alpha }\cdot q_{i}$$

where *α*_Τ_ and *α* are parameters that depend on the milling conditions and the properties of the material, respectively, while *x* is the particle size of size class *i*. *q*_i_ is a correction factor that defines the region of the breakage, i.e., normal breakage and abnormal breakage region. In the abnormal breakage region, particles are too large to be properly nipped by the media, and deviation from the first-order breakage occurs. *q*_i_ is calculated from Equation (4),

(4) $$q_{i}=\frac{1}{1+{\left(x/\mu \right)}^{\Lambda }}$$

where *μ* is a parameter that depends on milling conditions, and *Λ* is a positive parameter that indicates how fast the breakage rate decreases as feed size increases.

Austin et al. [12] defined primary breakage as the distribution of the particles produced before any of the particles are further broken inside the grinding mill. The set of primary particles produced can be represented by the breakage function *b*_i,j_, which is the mass fraction of size interval *j* broken into size interval *i*. The breakage function is usually represented in cumulative form, *B*_i,j_, which defines the mass fraction of material broken from size *j*, which appears less than the upper size of size interval *i*.

The values of *B*_i,j_ are estimated from the size distribution at short grinding times using as initial feed a narrow particle size fraction *j* (the one-size fraction BII method), as shown in Equation (5) [19],

(5) $$B_{i,j}=\frac{log[(1-P_{i}(0)/(1-P_{i}\left(t\right))]}{log[(1-P_{j+1}(0)/(1-P_{j+1}(t)]}$$

where *P*_i_(t) is the mass fraction less than size *x_i_* of size interval *i* at time *t*.

*B*_i,j_ can also be fitted to an empirical function (Equation (6)),

(6) $$B_{i,j}=\Phi_{j}\cdot {\left(\frac{x_{i-1}}{x_{j}}\right)}^{\gamma }+(1-\Phi_{j})\cdot {\left(\frac{x_{i-1}}{x_{j}}\right)}^{\beta }$$

where *x*_j_ is the top size of size interval *j*, *B*_i,j_ is the cumulative breakage function, and *Φ*_j_, *γ* and *β* are parameters that depend on the properties of the material; *Φ*_j_ and *β* show how rapidly fractions close to feed size are reduced to a lower size, while *γ* characterizes the relative mass of fines produced after breakage.

Industrial mills typically operate in a closed circuit with a classifier to prevent fine particles from entering the mill and control the size of the final product. In this respect, the effect of classification efficiency and circulating load on the efficiency of closed grinding circuits (i.e., its capacity to produce the desired final product) has been the subject of research for many years [20,21,22]. Among other classifiers, hydrocyclones are the most widely used in the mineral processing industry due to their simplicity and cost-saving [23]. However, one of the inherent limitations of hydrocyclone operation is its inefficiency in eliminating fine mineral particles in the coarse product, which affects mill performance [6].

Towards understanding the milling process, the effect of operating parameters on milling efficiency has been examined in recent years [24,25]. Other studies have reported that the breakage rate of particles varies significantly with the size distribution of the mill hold-up. In this respect, Austin and Bagga [19] investigated the dry grinding kinetics of several cement clinkers and two coals using a laboratory ball mill. They reported that the breakage rate of particles slows down due to the cushioning action of fines on the coarser particles during dry grinding. On the other hand, Gupta [26] analyzed the grinding of mixtures of coarse and fine fractions using limestone and quartz as test materials. He reported that the breakage rate of coarse particles increases with an increasing proportion of fine particles. Rajamani and Guo [27] performed wet grinding tests using limestone and estimated the breakage rates for all individual size classes. Using the G-H method proposed by Kapur [28], they observed that the breakage rate increased or decreased with time for all size classes, and this variation depended on the size distribution of the initial feed. Fuerstenau et al. [29] investigated the breakage rate of feeds consisting of mono-sized (10 × 14 mesh) dolomite with various proportions of fines (−100 mesh) after dry grinding tests in a ball mill. These researchers stated that the breakage rate of 10 × 14 mesh fraction increases as the amount of −100 mesh material in the feed increases. Similar results were observed in Verma and Rajamani’s study [30]. They reported that the hydrocyclone operation could be intentionally adjusted to recirculate some of the fines as the breakage rate of coarse material increases in the presence of fines.

The research discussed above was found to be limited to discovering the overall effect of the fines present in a mill on the breakage rate of coarse particles. In fact, these researchers have used mono-sized fractions and several proportions of fine material as the starting feed in the mill. According to Gupta [16], the size distributions generated by grinding mono-sized fractions are not representative of those obtained during industrial milling operations. In such lab-scale grinding environments, coarse particles grind at rates different from those observed in full-scale operations characterized by a natural-size distribution environment. Thus, this study aims to determine the breakage parameters in various natural-size distribution environments generated by closed-cycle grinding tests using different reference sieves. These natural-size distributions obtained after equilibrium conditions simulate industrial milling conditions. The Moly-Cop Tools™ v.1.0 software was used to back-calculate the breakage parameters obtained from the tests. Then, these parameters were entered into the MODSIM^TM^ software to simulate the closed-cycle grinding process.

## 2. Materials and Methods

In the present study, two materials, namely marble and quartzite, obtained from West Crete, Greece, were used. The marble samples are composed of calcite (95 wt.%) and dolomite (4 wt.%), while quartzite is composed of quartz (90 wt.%) and some mica (5 wt.%). The porosity and density determined with the use of the Archimedes method [31] were, respectively, 0.3% and 2.72 t/m^3^ for marble and 0.9% and 2.59 t/m^3^ for quartzite. Since marble’s hardness on the Mohs scale is much lower (ranging from 3 to 4) compared to that of quartzite (ranging from 7 to 8), it is expected that they will behave differently during grinding. Other properties affecting the grinding behavior of the test materials, e.g., uniaxial compressive strength and modulus of elasticity, are outlined in a previous study [32]. The received samples were homogenized by the cone and quarter method, and a representative amount was crushed to less than 3.35 mm using a Fritsch-type jaw crusher.

**Figure 1:**
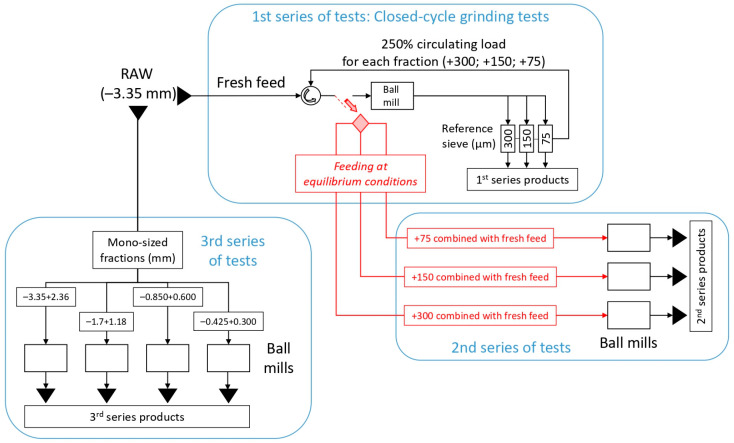
Flow chart of the experimental procedure.

Three series of grinding tests were carried out using a laboratory scale ball mill (D × L = 204 mm × 166 mm) that operated at *N* = 66 rpm (1.1 Hz), corresponding to 70% of its critical speed (Figure 1). The charge of the mill consisted of 77 balls of 25.4 mm diameter and density of 7.85 t/m^3^, corresponding to the ball filling volume *J* = 20%. The material filling volume was kept at *f*_c_ = 4% (215 mL), corresponding to 351.5 g and 342.5 g of marble and quartzite, respectively. The flow chart of the experimental procedure is shown in Figure 1.

In the first series, closed-cycle grinding tests were performed with a 250% circulating load to simulate industrial milling operations. According to the procedure followed, each initial feeding sample with a size less than 3.35 mm was fed into the mill and ground for an arbitrary number of mill revolutions (e.g., 50–100 revolutions, depending on the hardness of the material). After the first cycle, the product was sieved to the reference size, and the undersize was weighed and removed. The oversize plus an amount of fresh feed were combined to obtain the initial material volume filling of 4%. Then, this combined material was returned to the mill for the second grinding cycle. The weight of undersize produced per mill revolution (*Gpr*_1_) for the first cycle was calculated using Equation (7). For the second cycle, the number of required mill revolutions (*n*_2_) was calculated by considering 250% circulating load, according to Equation (8) [33].

(7) $${Gpr}_{1}=\frac{W_{p1}-\left(W_{F1}\cdot U\right)}{R_{1}}$$

(8) $$n_{2}=\frac{\left(\frac{W_{F1}}{3.5}\right)-\left(W_{P1}\cdot U\right)}{{Gpr}_{1}}$$

where *R*_1_ is the number of revolutions for the first cycle, *U* is the percentage of undersized material in the fresh feed, and *W*_F1_ and *W*_P1_ are the weights of the fresh feed and the product (undersized material) for the first cycle, respectively. The process was repeated until the weight of undersize produced per revolution (*Gpr*) reached the steady state (equilibrium) conditions in the three last cycles. The final value of *Gpr* was calculated as the average of the last three cycles. It is mentioned that the closed-cycle grinding tests of the first series were conducted using three specific reference sieves, namely 300 µm, 150 µm and 75 µm. Then, the particle size distributions (PSDs) of the final products at equilibrium conditions were determined using a Malvern type S Mastersizer (particle size range between 0.05 and 850 μm) and laser diffraction (LD) technique. The resulting PSDs were evaluated and compared with respect to the material type and reference sieve used. It is also noted that since the sieving procedure was used to determine the particle size distributions of the feeding materials, the resulting product size distributions were corrected for comparison purposes using the apparent shape factor (ASF) calculated for each material, as described in a previous study [34].

For the second series, the ground product of the final cycle was sieved again to each reference size (300 µm, 150 µm and 75 µm), and the undersize was weighed and removed. Then, an equal mass of fresh feed was added to the oversize (circulating load), and the combined material was used for the tests. This combined material can be considered as the feed to the industrial mill after equilibrium conditions are reached. As a result, three different feeding materials (abbreviated as EFMs) were generated for marble and quartzite, and their grinding behavior was investigated based on kinetic modeling approaches. For this purpose, grinding tests were carried out for various grinding times, and the breakage rate of the top-size fraction, i.e., −3.35 + 2.36 mm, was determined for the two types of material. Therefore, these tests enable the evaluation of the breakage rate parameters for materials with natural-size distributions obtained after equilibrium conditions, thereby simulating industrial grinding systems.

In the third series of tests, the initial samples of the two types of materials (particle size less than 3.35 mm) were sieved using a series of sieves to prepare four mono-sized fractions, namely −3.35 + 2.36 mm, −1.7 + 1.18 mm, −0.850 + 0.600 mm and −0.425 + 0.300 mm. Then, tests were carried out for various grinding times under the same conditions followed in the previous tests, i.e., *N* = 66 rpm, *J* = 20% and *f*_c_ = 4%. The products obtained after each grinding time were wet-sieved using a series of screens for the determination of PSD. The results of breakage analysis were examined and compared with those obtained when EFMs were used as feeding materials to the mill.

The specific surface area (SSA) of the grinding products was measured using both LD and Brunauer–Emmett–Teller (BET) nitrogen adsorption techniques. LD uses the Mia theory of light scattering to determine PSDs, assuming a volume-equivalent sphere model, whereas BET measures the actual surface area of solids based on the physical adsorption of gas molecules on their surface [35,36]. According to the LD technique, the SSA of sample materials is estimated using Equation (9),

(9) $$S_{w}=\left(\frac{f}{k}\right)\cdot \frac{1}{\rho_{p}\cdot D\left[3,2\right]}$$

where *S*_w_ is the specific surface area, *ρ*_p_ is the particle density, D[3,2] is the mean surface area (Sauter mean diameter), and *f*, *k* are the surface and volume coefficients (for spheres f/k = 6).

The power of the mill, *P* (kW), was calculated using Equation (10), proposed by Bond [37,38],

(10) $$P=7.33\cdot J\cdot N_{r}\cdot (1-0.937\cdot J)\cdot \rho_{b}\cdot L\cdot D^{2.3}\cdot \left(1-\frac{0.1}{2^{9-10\cdot N_{r}}}\right)$$

Once the power of the mill over a grinding period *t* is calculated, the specific energy *ε* (kJ/kg) (energy input *E* (kJ) per unit mass *m* (kg) of the feed) can be defined as

(11) $$\varepsilon =\frac{E}{m}=\frac{P\cdot t}{m}$$

Regarding the closed-cycle grinding tests, *ε* (kJ/kg) is calculated by taking into consideration the average weight of undersize produced per second (*Gps*) of the last three cycles, according to Equation (12),

(12) $$\varepsilon =\frac{P}{Gps}$$

The breakage parameters, i.e., *α*_T_, *α*, *μ*, *Λ*, *Φ*_j_, *γ* and *β*, were back-calculated using the Moly-Cop Tools™ v.1.0 software. The software includes a set of easy-to-use Excel spreadsheets that use all the differential equations of the population balance model for the calculation of *S*_i_ and *B*_i,j_ parameters [15]. The simulation of the closed-cycle grinding process was carried out using MODSIM^TM^ academic version 3.6.25 [39,40]. MODSIM^TM^ is based on population balance modeling and allows the simulation of integrated mineral processing circuits. It has been widely used for process design and optimization and as an academic tool as well. Also, this simulator can up-scale results to a full-scale mill, and therefore, the breakage parameters determined in the laboratory batch test can be used as input data.

## 3. Results and Discussion

### 3.1. First Series of Tests

#### 3.1.1. Examining the Fitting Accuracy of the Particle Size Distribution Models

**Figure 2:**
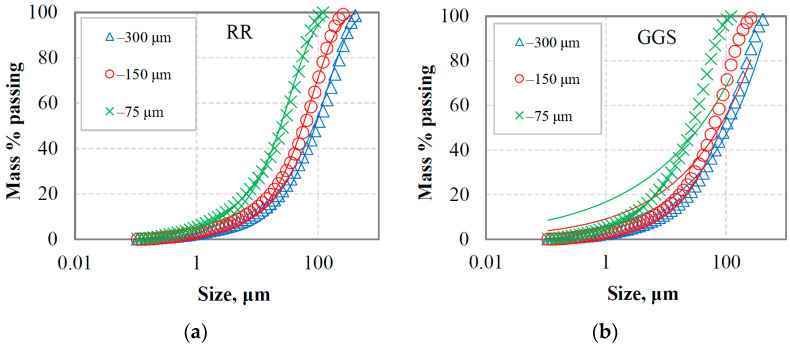
Particle size distributions of marble final grinding products at equilibrium conditions for three reference sieves using (**a**) Rosin–Rammler (RR) and (**b**) Gates–Gaudin–Schuhmann (GGS) distribution models. Solid lines denote the model data.

**Figure 3:**
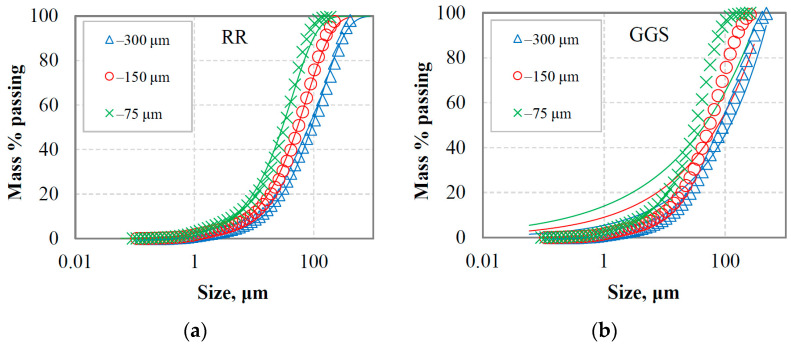
Particle size distributions of quartzite final grinding products at equilibrium conditions for three reference sieves using (**a**) Rosin–Rammler (RR) and (**b**) Gates–Gaudin–Schuhmann (GGS) distribution models. Solid lines denote the model data.

Figure 2a,b and Figure 3a,b present the PSDs of the final products for marble and quartzite, respectively, at equilibrium conditions when three reference sieves were used, namely 300, 150 and 75 μm. The mathematical simulation of the grinding products was carried out using the Rosin–Rammler (RR) and Gates–Gaudin–Schuhmann (GGS) distribution models [41]. The mathematical description of these models can also be found in a previous study [42]. In this regard, non-linear least square fitting from the Solver tool of Microsoft Excel was used, and the model parameters were estimated. Models comparison was carried out using evaluation indices, i.e., root mean square error (RMSE), adjusted correlation coefficient (R^2^ adj.) and standard error (SE) [43]. As seen in these figures, models have significantly different fitting capabilities; RR is a particularly suitable model to describe the PSD of the final products, while GGS provides very low accuracy. This is consistent with the results of Table 1, which show that R^2^ adj. ranged from 0.998 to 0.999 and 0.884 to 0.962 when, respectively, RR and GGS models were fitted to the experimental data. The highest fitting accuracy of RR was also indicated by the lower RMSE (ranging from 1.32 to 2.52) and SE (ranging from 1.22 to 2.15) values; the respected values for the GGS model ranged from 7.21 to 15.02 and 6.22 to 14.55.

RR and GGS are considered the two most common distribution models that are often used to describe the PSDs in mineral processing operations. However, GGS is often preferred in certain applications, such as coal processing, while the RR is useful for monitoring grinding operations for highly skewed distributions [44,45]. According to Taşdemir and Taşdemir [46], the GGS model describes the PSDs obtained by low-energy events better, i.e., jaw and cone crushing, while the RR model is more suitable for PSDs obtained by high-energy events, i.e., hammer crushing and ball milling.

**Figure 4:**
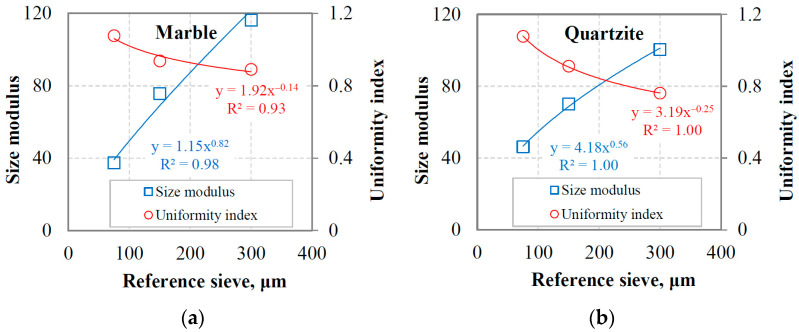
Size modulus and uniformity index versus reference sieve using the RR model for (**a**) marble and (**b**) quartzite.

The RR model parameters, i.e., size modulus and distribution modulus (uniformity index), were also estimated with respect to the reference sieve to characterize the PSDs of the final products for marble and quartzite at equilibrium conditions. As shown in Figure 4a,b, the size modulus increases, while the uniformity index decreases with the increase in the reference sieve size. These results indicate that the finer the product size, the higher the uniformity index value, and the PSD becomes narrower. Very strong correlations (R^2^ values are greater than 0.93 and equal to 1.00 for marble and quartzite, respectively) are obtained with the use of exponential functions between size modulus or uniformity index and the reference sieve size. In industrial operations, ball mills are usually in closed circuits with a classifier, and the interaction between them affects the grinding-product size when equilibrium conditions are reached. Also, closed-cycle laboratory grinding tests that simulate industrial milling conditions use reference sieves that play the role of the industrial classifier. Therefore, these correlations (Figure 4) allow for investigating how the grinding product size changes when considering different laboratory reference sieve sizes or cut sizes in industrial operations. Magdalinović [47] performed locked-cycle grinding tests following the Bond test procedure [37,48]. The materials used were copper ore, andesite and limestone. He stated that a linear relationship exists between the reference sieve size and the 80% passing size of the grinding product after equilibrium conditions are reached. Despite using different materials and test conditions, his results are relatively close to those of the present study.

#### 3.1.2. Calculation of Specific Surface Area and Energy Efficiency

**Figure 5:**
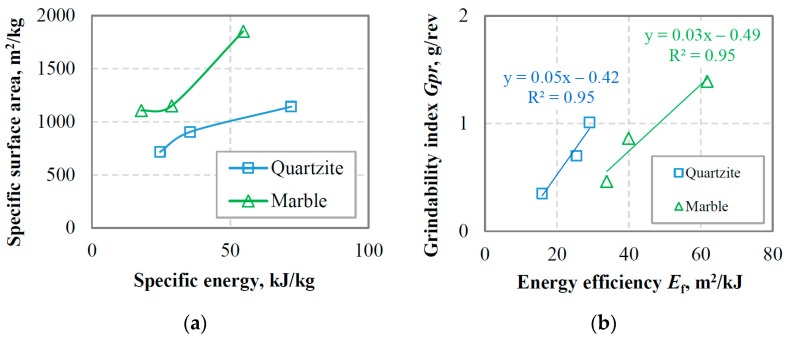
(**a**) Variation in BET-specific surface area with specific energy; (**b**) grindability index versus energy efficiency.

Table 2 shows the specific surface area of the grinding products at equilibrium conditions determined by either BET or LD techniques for marble and quartzite. For each grinding product, the specific energy consumption *ε* was also calculated using Equation (12). The median size *d*_50_ was determined using Equation (9) and assuming spherical particle shape (f/k = 6). It is shown that the energy consumption *ε* for quartzite is higher than for marble. This can be attributed to the difference in hardness between the two materials (3–4 for marble and 7–8 for quartzite on the Mohs scale). Regarding the SSA, the results show that the BET technique, which takes into account various factors, including the geometry of pores and the presence of cracks, provides, in all cases, a much higher SSA compared to that obtained with the LD technique. In this respect, BET is regarded as the most fundamental bulk surface area measurement technique; however, it is time-consuming and requires meticulous sample preparation [49,50]. By comparing the two materials tested, it is evident that the SSA of the grinding products of marble compared to that of quartzite is higher when considering the same reference sieve size. This is explained by the fact that marble produces the largest amount of fine particles, which significantly contributes to SSA. As also seen in Figure 5a, the SSA (BET) of marble grinding products increases sharply from 1105 to 1852 m^2^/kg when the specific energy increases from 17.9 to 54.7 kJ/kg, while a gradually increasing trend was observed for quartzite. As a result, the energy efficiency (*E*_f_), defined as the SSA per unit of specific energy consumption (slope at each point of the curves), is affected by the type of material tested. In this respect, the energy efficiency for marble grinding is 57 to 113% higher than that of quartzite. The weight of undersize produced per revolution at equilibrium conditions (referred to as grindability index, *Gpr*) as a function of energy efficiency *E*_f_ is shown in Figure 5b. It is observed that strong correlations (R^2^ = 0.95) between *Gpr* and *E*_f_ are obtained with the use of linear functions. The results show that for the production of a certain amount per mill revolution, marble exhibits a higher energy efficiency compared to quartzite. However, the rate of production (as observed from the slope of the straight lines) for quartzite is more pronounced. This is in line with previous findings that excess fine particles produced during marble grinding enhance the cushioning effect and reduce grinding efficiency [51].

### 3.2. Second Series of Tests

#### 3.2.1. PSDs of the Equilibrium Feeding Materials

**Figure 6:**
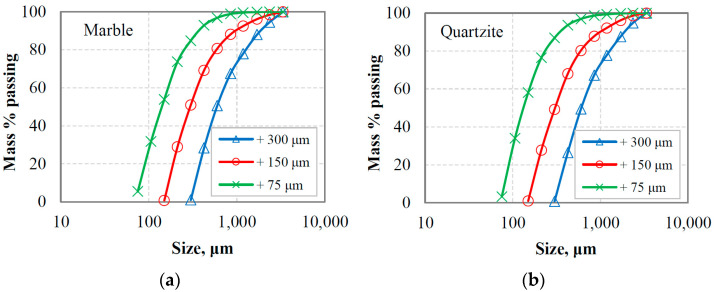
PSDs of the oversize (circulating load) at equilibrium conditions for (**a**) marble and (**b**) quartzite.

**Figure 7:**
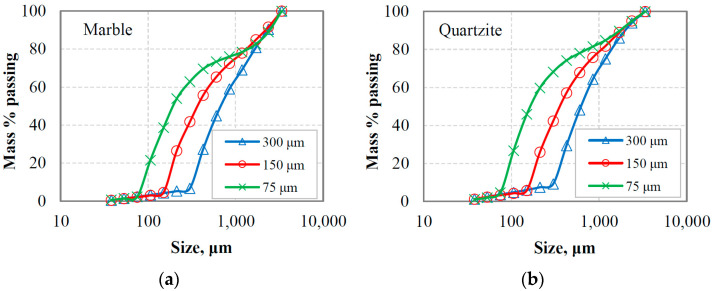
PSDs of the equilibrium feeding materials for (**a**) marble and (**b**) quartzite.

Figure 6a,b present the PSDs of the oversized material (or circulating load) after the last cycle where equilibrium conditions were reached for marble and quartzite, respectively. The circulating loads obtained using three reference sieve sizes (300 µm, 150 µm and 75 µm) were combined with the corresponding fresh feeds, and the generated mixture materials (referred to as equilibrium feeding materials, EFMs) were subjected to short grinding time tests to study the grinding kinetics. As seen in Figure 7a,b, the greater percentage of the feeding materials are coarser than the reference sieve size, while a small amount of fine particles originating from the fresh feeds is observed.

**Figure 8:**
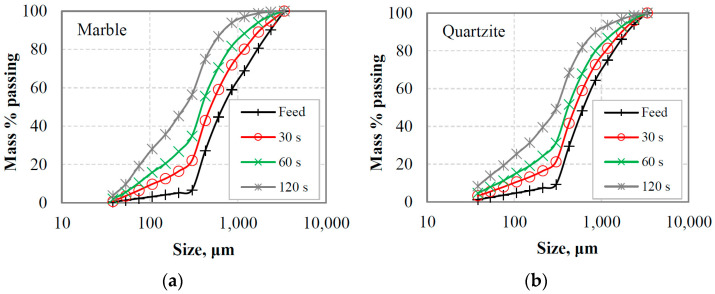
Evolution of PSDs of the equilibrium feeding materials after short grinding times for (**a**) marble and (**b**) quartzite; the reference sieve size used was 300 μm.

**Figure 9:**
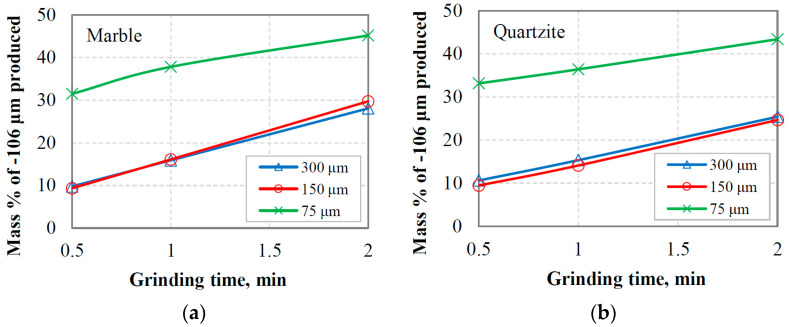
Mass % of −106 μm fraction produced vs. grinding time when different reference sieve sizes were used in closed-cycle grinding tests for (**a**) marble and (**b**) quartzite.

The PSDs of the equilibrium feeding materials and the products as a function of grinding time for the two materials tested are shown in Figure 8a,b. These figures, which are used as an example, correspond to the reference sieve size of 300 μm. It is obvious that more fine particles are produced as grinding proceeds, and the product particle size distribution curves acquire a similar shape. However, among other factors, such as the type of material and operating conditions [52], the feed size distribution in batch grinding affects the shape of curves and the production of fine particles. In this respect, Figure 9a,b present the mass % of −106 μm fraction produced as a function of grinding time when different reference sieve sizes (300 µm, 150 µm and 75 µm) were used in closed-cycle grinding tests. It is shown that the use of either 300 or 150 μm reference sieve in closed-cycle tests results in essentially the same production of fine particles for both materials tested. Also, the slope of the plots (Figure 9a,b), which indicates the production rate of fines, is slightly higher when using 300 or 150 μm reference sieve in closed-cycle tests compared to that of the 75 μm sieve. This shows that feed size distributions with varying proportions of fines can affect the breakage rate of particles. Given that the increase in breakage rates in industrial production processes has important implications, it is considered crucial to investigate the effect of fines content on the grinding kinetics.

#### 3.2.2. Modeling of Grinding Kinetics of Equilibrium Feeding Materials

**Figure 10:**
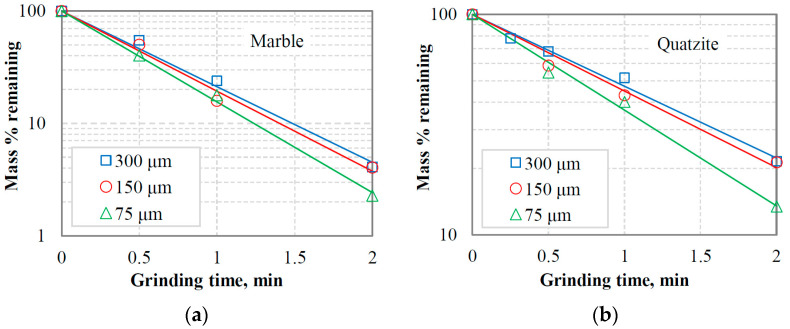
First-order plots of the mass % remaining in the −3.35 + 2.36 mm top-size class vs. grinding time in equilibrium feedings of closed-cycle tests for (**a**) marble and (**b**) quartzite when different reference sieve sizes were used.

Figure 10a,b show in normal-log plots the mass % remaining in the −3.35 + 2.36 mm fraction as a function of grinding time for marble and quartzite under the assumption that grinding obeys a first-order law, i.e., the breakage rate *S*_i_ is independent of time [12]. This fraction represents the top-size class of the equilibrium feeding materials in closed-cycle grinding tests and the effect of the reference sieve size (300 μm, 150 μm or 75 μm) on the breakage rate *S*_i_ (min^−1^), obtained from the slopes of the straight lines, was investigated. It is evident that the breakage rate of the top-size class of the feeding materials increases as the aperture size of the selected reference sieve decreases, and consequently, the content of fines in the mill increases. The results are consistent with earlier research [29,53], which showed that the presence of fines in the mill increases the breakage rate of the coarse fraction, resulting in more efficient grinding. However, in those studies, the feeds to the mill consisted of mixtures of coarse fractions and fines in various proportions, in contrast to this study, where feed size distributions of closed-cycle grinding tests at equilibrium conditions were used.

**Figure 11:**
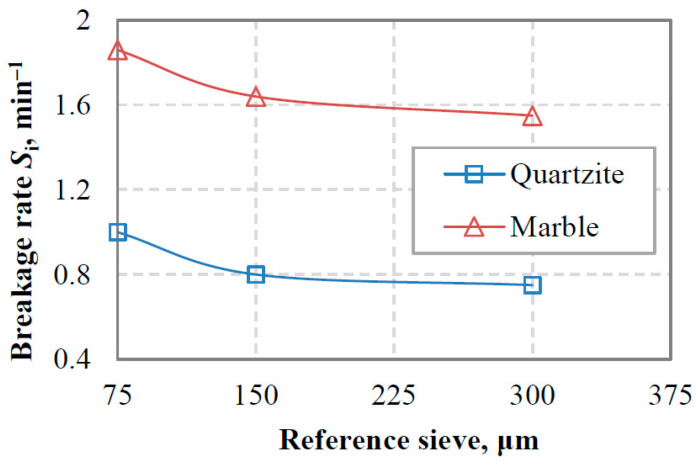
Variation of breakage rates of the −3.35 + 2.36 mm size class with reference sieve size in marble and quartzite closed-cycle equilibrium feedings.

Figure 11 shows the variation in breakage rates of the −3.35 + 2.36 mm size class with reference sieve size for the closed-cycle equilibrium feedings of marble and quartzite. It is observed that the breakage rate values of the top-size class of marble are higher than those of quartzite. The marble breakage rate was 1.55 min^−1^ using the 300 µm reference sieve and reached 1.86 min^−1^ when the 75 µm sieve size was used; an increase of 20% was observed. Regarding the top-size class of quartzite, its breakage rate increased by 33.3%, from 0.75 min^−1^ to 1.0 min^−1^, when the reference sieve size was reduced from 300 µm to 75 µm. The small increase in the breakage rate of the top-size class of marble can be attributed to the cushioning effect of fines on the coarse particles when their amount exceeds certain limits [19,51]. In this case, the impact forces are ineffective, and the breakage rate is reduced.

### 3.3. Third Series of Tests

#### 3.3.1. Determination of Breakage Parameters

**Figure 12:**
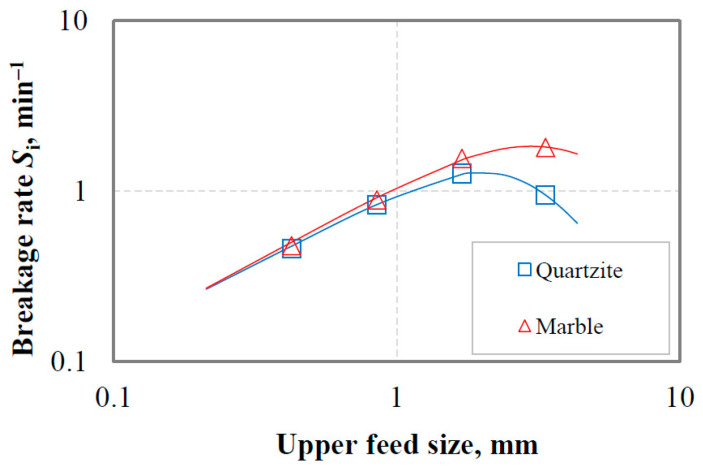
Experimental (symbols) and modeled (solid lines) variation of breakage rate *S*_i_ as a function of the upper feed size for marble and quartzite.

In this series of tests, four mono-sized fractions, namely −3.35 + 2.36 mm, −1.7 + 1.18 mm, −0.850 + 0.600 mm and −0.425 + 0.300 mm, were ground in a laboratory ball mill for various grinding times, i.e., 0.5, 1, 2 and 4 min. Since grinding exhibits first-order behavior, the breakage rate *S*_i_ was determined from the first-order plots of the mass (%) remaining vs. grinding time for different mono-sized feed fractions. The determined *S*_i_ values for each feed fraction were used in Equations (3) and (4), and the kinetic parameters, i.e., *α*_T_, *α*, *μ* and *Λ*, were calculated using a non-linear regression technique. This technique finds the best-fit values of the model parameters by minimizing the square of the differences between the experimental and the predicted values. Figure 12 shows the evolution of the *S*_i_ values versus the upper feed size on a log–log scale, while Table 3 presents the values of breakage rate parameters for each material tested. Figure 12 shows that the variation of breakage rate values with feed size follows a known trend for both materials. *S*_i_ increases with increasing feed size up to a certain particle size (referred to as the optimum size) and then drops as the size becomes coarser because the particles are too big to be properly nipped and broken by the grinding media used [54,55]. Thus, for each material, there is an optimum feed size (*x*_m_) at which the breakage rate obtains its maximum value (*S*_m_). The *x*_m_, *S*_m_ values are 2.98, 1.83 and 2.00, 1.29 for marble and quartzite, respectively. The values of the parameters shown in Table 3, namely *S*_i/3.35_ (i.e., the breakage rate of −3.35 + 2.36 mm fraction) and *α*_Τ_, show that marble is ground at a higher rate than quartzite.

**Figure 13:**
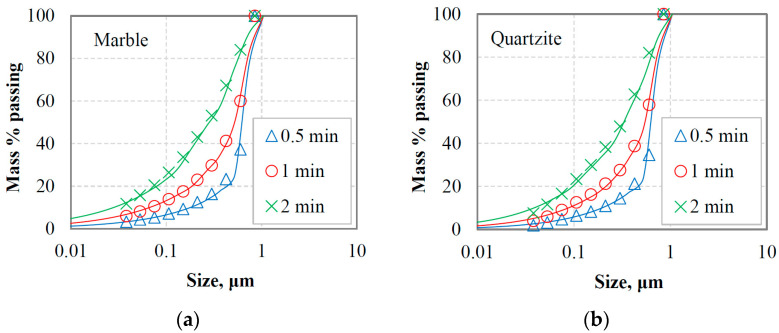
Experimental (symbols) and reproduced (solid lines) PSDs at different grinding times, using the back calculated breakage parameters, for the −0.850 + 0.600 mm fraction of (**a**) marble and (**b**) quartzite.

The breakage function *B*_i_,_j_ parameters, i.e., *Φ*_j_, *γ* and *β*, were calculated from the size analysis of the products at short grinding times. According to the one size fraction BII method, the values of *B*_i_,_j_ can be determined when a narrow particle size fraction is used as feed material to the mill and the selected grinding time results in a 20% to 30% material that is broken out from the top-size fraction [12]. In this respect, *B*_i_,_j_ values were determined by fitting the experimental data to Equation (5), while the *Φ*_j_, *γ* and *β* parameters were determined from Equation (6) using a non-linear regression technique. It is also stated that a back-calculation method with Moly-Cop Tools™ v.1.0 software was employed to determine the breakage parameters shown in Table 3. Three size distributions (−1.7 + 1.18 mm, −0.850 + 0.600 mm and −0.425 + 0.300 mm) were used as input in Moly-Cop Tools™ at three grinding times (0.5, 1 and 2 min), and the best combination of *α*_T_, *α*, *μ*, *Λ*, *Φ*_j_, *γ* and *β* was determined. In order to obtain accurate estimates, the number of parameters was reduced by keeping constant those generally considered to be characteristic of the material, i.e., *α* and *β*. Then, other parameters that give the best fit between experimental and reproduced size distributions were calculated. Figure 13a,b compare the experimental and reproduced size distributions of marble and quartzite obtained at different grinding times for the −0.850 + 0.600 mm feed size. The results indicate that very good fitting curves are obtained for the −0.850 + 0.600 mm feed fraction when the breakage parameters derived from the Moly-Cop Tools™ (Moly-cop Chile S.A., Santiago, Chile) were used. The R^2^ adj. ranged from 0.996 to 0.999 for both materials tested. A very good match was also observed between the experimental and predicted PSDs for the other feed fractions, namely −1.7 + 1.18 mm and −0.425 + 0.300 mm.

#### 3.3.2. Effect of Fines Accumulation on Breakage Rate of Coarse Particles

**Figure 14:**
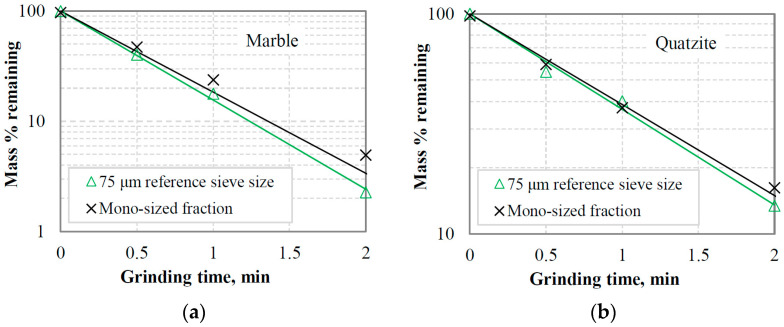
First-order plots of the mass % remaining in the −3.35 + 2.36 mm size class (mono-size fraction) vs. grinding time for (**a**) marble and (**b**) quartzite. For comparison, the first-order plots of the top-size class in equilibrium feedings of closed-cycle tests using a reference sieve size of 75 μm are also shown.

Figure 14a,b show the first-order plots of the mass % remaining in the mono-size fraction −3.35 + 2.36 mm as a function of grinding time for the materials tested. These figures also show, for comparison, the first-order plots of the top-size class −3.35 + 2.36 mm in equilibrium feedings of closed-cycle tests when a reference sieve size of 75 μm was used. The results show that the breakage rates *S*_i_ of the mono-size fraction −3.35 + 2.36 mm, obtained from the first-order plots, are lower than those of the same fraction (top-size class) in closed-cycle tests when a 75 µm reference sieve was used. The breakage rates for the top-size class of marble and quartzite are 1.86 and 1.00 min^−1^, respectively, while the corresponding rates for the mono-size fractions are 1.69 and 0.95 min^−1^. This indicates that the presence of fines, particularly for marble, is beneficial for grinding coarse fractions. However, as was previously noted, an excessive amount of fine particles in the mill enhances the cushioning action. Nevertheless, the results should raise questions about effective grinding in closed-circuit operations. In these operations, the mill is in a closed circuit with a classifier, and the goal is to increase the circuit’s capacity while minimizing the amount of fines that are returned to the mill. However, the reduction in fines in the mill may result in a decrease in the breakage rate [27,29,53], which would increase the circulating load. The latter has the effect of reducing the circuit capacity. The importance of classification in closed-cycle grinding operations has been reported in several previous studies [56,57,58].

### 3.4. Simulation of the Closed-Cycle Grinding

**Figure 15:**
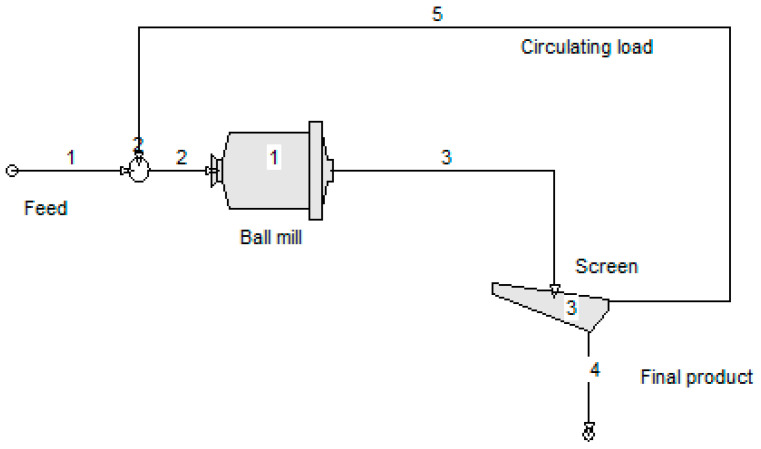
Flow chart of simulated closed-cycle grinding implemented in the MODSIM^TM^ simulator.

The simulation of the closed-cycle grinding for marble and quartzite was performed using the MODSIM^TM^ simulator. Figure 15 shows the flow chart implemented in this simulator. The unit model MILL available in MODSIM^TM^ was selected as the mill model. MILL is the simplest model for the ball mill, which is based on standard Austin’s models (Equations (3)–(6)) and does not need any details of the mill geometry. Thus, the breakage parameters, i.e., *α*_T_, *α*, *μ*, *Λ*, *Φ*_j_, *γ* and *β*, determined by the back-calculation method using the Moly-Cop Tools™ (Table 3), were used as initial input in the unit model MILL of the simulator. The mean residence time of the particles in the mill must also be given. Regarding the screen, the model SCRN was used to simulate the closed-cycle grinding. In this model, three variables are required, namely screen opening, efficiency (90% is used as default) and surface water % on screen oversize.

**Figure 16:**
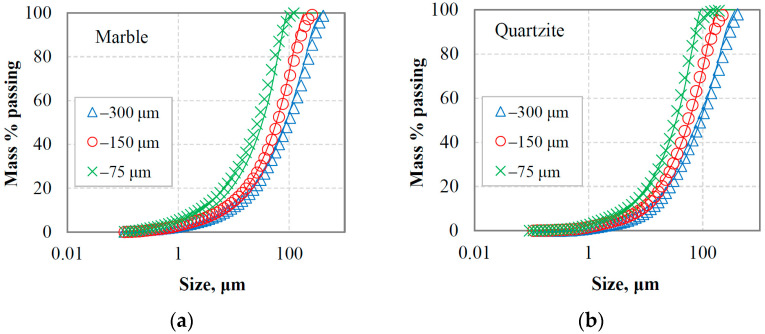
Comparison of experimental (symbols) and simulated (solid lines) product PSDs for (**a**) marble and (**b**) quartzite when different reference sieve sizes were used in MODSIM^TM^ simulator.

According to the procedure, the feed size distribution to the circuit was entered in MODSIM^TM^, and the product PSDs obtained from the simulation were compared with the experimental ones. Firstly, the residence time of the particles in the mill that gives the best fit between the experimental and the reproduced size distributions of the final products was determined. In this regard, the optimum residence time of 2.2 min was determined in the case of marble when a reference sieve of 300 μm was used in the closed-cycle grinding simulation. The simulation was then validated by the results obtained using other reference sieves, i.e., 150 and 75 μm, keeping all input parameters constant. Figure 16a,b show the simulation results using the MODSIM^TM^ simulator compared to the experimental data obtained from the laboratory grinding tests for marble and quartzite. It is clearly shown that the simulator could predict the product PSDs in closed-cycle grinding operations with high accuracy. This is consistent with the high R^2^ adj. values determined; R^2^ adj. was in the range of 0.995 to 0.999 for both materials tested.

## 4. Conclusions

In this study, closed-cycle grinding tests were performed to examine the effect of the size-distribution environment on the breakage parameters of two test materials, namely marble and quartzite. These tests evaluated and compared the final products under equilibrium conditions with respect to the material type and reference sieve utilized.

Regarding the evaluation of the final products, it was found that the RR distribution model could accurately characterize the PSDs, whereas the GGS provides very low accuracy. The adjusted correlation coefficient (R^2^ adj.) ranged from 0.998 to 0.999 and 0.884 to 0.962 when the RR and GGS models, respectively, were fitted to the experimental data. In addition, it was revealed that the size modulus or uniformity index of the RR model is very well correlated (R^2^ values are greater than 0.93) with the reference sieve size. Based on the BET-specific surface area measurements of the grinding products and energy consumption, the energy efficiency for marble was found to be 57 to 113% higher compared to quartzite. The grindability index (g/rev) was also very well correlated (R^2^ = 0.95) with the energy efficiency for both materials tested using linear functions.

When the equilibrium feeding materials (EFMs) obtained from different reference sieve sizes were subjected to short-time grinding tests, it was found that the size-distribution environment affects the breakage rate of particles. The use of 300 or 150 μm reference sieves resulted in a slightly higher production rate of fines (−106 μm) than the use of a 75 μm sieve. In addition, it was found that the breakage rate of the top-size class (−3.35 + 2.36 mm) of the EFMs increases with decreasing sieve aperture size, indicating the beneficial effect of fines on milling efficiency. In general, the breakage rate values for marble were higher compared to quartzite. However, in the case of marble, a smaller increase (20% vs. 33.3%) with a decreasing sieve aperture size was observed, and this was attributed to the cushioning effect when fines exceeded certain limits.

Based on the one-size fraction BII method, it was shown that the variation of breakage rate values with feed size follows a well-known trend for the test materials. The breakage rate increases with increasing feed size up to a specific size, but above this size, it decreases sharply. By comparing the first-order plots of the top-size class (−3.35 + 2.36 mm) of EFS with the mono-size fraction −3.35 + 2.36 mm, it was found that the breakage rate of this size class is higher for the feeds generated from the closed-cycle tests, for both materials tested. This also indicates the increase in grinding efficiency when fine particles are present inside the mill.

Regarding the simulation of the closed-cycle grinding for marble and quartzite, it was found that the back-calculated breakage parameters derived by the Moly-Cop Tools™ software could be reliably used as initial input in the MODSIM^TM^ simulator. It was confirmed that for both materials, MODSIM^TM^ could be a valuable tool for the simulation of closed-cycle grinding operations, as a very good agreement was observed between the experimental and simulated particle size distributions of the final products. The approach can also be used to scale up laboratory results to larger-scale grinding operations and study several factors affecting the process, thereby reducing the cost of experimentation and optimization. Future studies should aim to predict the results of pilot and full-scale grinding circuits under different operating conditions based on data from laboratory-scale grinding tests.

## Figures and Tables

**Table 1:** Statistical analysis of the accuracy of the fitting particle size distribution models (RR and GGS) on the final grinding products for marble and quartzite at equilibrium conditions.

Model	Index	Marble			Quartzite		
		−300 μm	−150 μm	−75 μm	−300 μm	−150 μm	−75 μm
RR	R^2^ adj.	0.997	0.998	0.998	0.999	0.999	0.997
	RMSE	2.52	1.91	2.08	1.38	1.32	2.25
	SE	2.00	1.71	1.87	1.33	1.22	2.15
GGS	R^2^ adj.	0.967	0.931	0.884	0.962	0.918	0.878
	RMSE	6.45	10.29	14.39	7.21	11.57	15.02
	SE	6.22	9.95	13.94	6.87	11.16	14.55

**Table 2:** Specific surface area and energy efficiency of the final products at equilibrium conditions.

Material	Product Size	BET	LD	d_50_	ε	E_f_
	μm	m^2^/kg	m^2^/kg	μm	kJ/kg	m^2^/kJ
Marble	−300	1105	245	96.2	17.9	61.8
	−150	1149	296	61.2	28.8	39.9
	−75	1852	556	26.7	54.7	33.8
Quartzite	−300	716	127	91.9	24.7	29.1
	−150	905	202	55.0	35.5	25.4
	−75	1142	313	32.4	72.0	15.9

**Table 3:** Values of breakage parameters for each material tested.

Material	*S*_i/3.35_	*α*_Τ_	*α*	*μ*	*Λ*	*Φ*_j_	*γ*	*β*
	min^−1^	min^−1^		mm				
Marble	1.81	1.09	0.90	3.71	3.10	0.63	0.73	3.30
Quartzite	0.95	0.88	0.84	2.76	3.15	0.73	0.85	3.30

## Data Availability

The data presented in this study are available on request from the author.

## References

[1] Pural Y., Çelik M., Ozer M., Boylu F. (2022). Effective circulating load ratio in mill circuit for milling capacity and further flotation process—Lab scale study. Physicochem. Probl. Miner..

[2] Petrakis E., Varouchakis E., Komnitsas K. (2023). Reliability of the non-linear modeling in predicting the size distribution of the grinding products under different operating conditions. Min. Metall. Explor..

[3] Peltoniemi M., Kallio R., Tanhua A., Luukkanen S., Perämäki P. (2020). Mineralogical and surface chemical characterization of flotation feed andproducts after wet and dry grinding. Miner. Eng..

[4] Jeswiet J., Szekeres A. (2016). Energy Consumption in Mining Comminution. Procedia CIRP.

[5] Morrell S. (2022). Helping to reduce mining industry carbon emissions: A step-by-step guide to sizing and selection of energy efficient high pressure grinding rolls circuits. Miner. Eng..

[6] Whitworth A.J., Forbes E., Verster I., Jokovic V., Awatey B., Parbhakar-Fox A. (2022). Review on advances in mineral processing technologies suitable for critical metal recovery from mining and processing wastes. Clean. Eng. Technol..

[7] Hodgkinson J.H., Smith M.H. (2021). Climate change and sustainability as drivers for the next mining and metals boom: The need for climate-smart mining and recycling. Resour. Policy.

[8] Stamboliadis E.T. (2004). Energy distribution in comminution: A new approach to the laws of Rittinger, Bond, and Kick. Can. Metall. Q..

[9] Saldaña M., Gálvez E., Navarra A., Toro N., Cisternas L.A. (2023). Optimization of the SAG Grinding Process Using Statistical Analysis and Machine Learning: A Case Study of the Chilean Copper Mining Industry. Materials.

[10] Lynch A.J. (1977). Mineral Crushing and Grinding Circuit—Their Simulation, Optimisation, Design and Control.

[11] Herbst J.A., Fuerstenau D.W. (1980). Scale-up procedure for continuous grinding mill design using population balance models. Int. J. Miner. Process..

[12] Austin L.G., Klimpel R.R., Luckie P.T. (1984). Process Engineering of Size Reduction: Ball Milling.

[13] Ipek H., Göktepe F. (2011). Determination of grindability characteristics of zeolite. Physicochem. Probl. Miner. Process..

[14] Gupta V.K., Sharma S. (2014). Analysis of ball mill grinding operation using mill power specific kinetic parameters. Adv. Powder Technol..

[15] Petrakis E., Stamboliadis E., Komnitsas K. (2017). Identification of optimal mill operating parameters during grinding of quartz with the use of population balance modelling. Kona Powder Part. J..

[16] Gupta V.K. (2019). An appraisal of the energy-size reduction relationships for mill scale-up design. Adv. Powder Technol..

[17] Austin L.G., Luckie P.T. (1972). Methods for determination of breakage distribution parameters. Powder Technol..

[18] Klimpel R.R., Austin L.G. (1977). The back-calculation of specific rates of breakage and non-normalized breakage distribution parameters from batch grinding data. Int. J. Miner. Process..

[19] Austin L.G., Bagga P. (1981). An analysis of fine dry grinding in ball mills. Powder Technol..

[20] Hukki R.T. (1979). Fundamentals of the closed grinding circuit. Eng. Min. J..

[21] Morrell S. (2008). A method for predicting the specific energy requirement of comminution circuits and assessing their energy utilisation efficiency. Miner. Eng..

[22] Jankovic A., Valery W. (2013). Closed circuit ball mill—Basics revisited. Miner. Eng..

[23] Tian J., Ni L., Song T., Olson J., Zhao J. (2018). An overview of operating parameters and conditions in hydrocyclones for enhanced separations. Sep. Purif. Technol..

[24] Abdelhaffez G.S., Ahmed A.A., Ahmed H.M. (2022). Effect of grinding media on the milling efficiency of a ball mill. Rud. Geol. Naft. Zb..

[25] Mannheim V., Kruszelnicka W. (2023). Relation between Scale-Up and Life Cycle Assessment for Wet Grinding Process of Pumice. Energies.

[26] Gupta V.K., Leschonski K. An appraisal of the linear first order kinetic model based ball mill design correlations. Proceedings of the World Congress Particle Technology, Part II: Comminution, (6. European Symposium on Comminution).

[27] Rajamani R.K., Guo D. (1992). Acceleration and deceleration of breakage rates in wet ball mills. Int. J. Miner. Process..

[28] Kapur P.C. (1982). An improved method for estimating the feed-size breakage distribution functions. Powder Technol..

[29] Fuerstenau D.W., Abouzeid A.M., Phatak P.B. Effect of fine particles on the kinetics and energetics of grinding coarse particles. Proceedings of the 119th AIME Annual Meeting, Salt Lake City.

[30] Verma R., Rajamani R.K. (1995). Environment dependent breakage rates in ball milling. Powder Technol..

[31] (2017). Natural Stone Test Methods. Determination of Real Density and Apparent Density, and of Total and Open Porosity.

[32] Petrakis E., Komnitsas K. (2018). Correlation between material properties and breakage rate parameters determined from grinding tests. Appl. Sci..

[33] García G.G., Oliva J., Guasch E., Anticoi H., Coello-Velázquez A.L., Menéndez-Aguado J.M. (2021). Variability Study of Bond Work Index and Grindability Index on Various Critical Metal Ores. Metals.

[34] Petrakis E., Komnitsas K. (2019). Effect of energy input in a ball mill on dimensional properties of grinding products. Min. Metall. Explor..

[35] Li M., Wilkinson D., Patchigolla K. (2005). Comparison of particle size distributions measured using different techniques. Part. Sci. Technol..

[36] Kuila U., Prasad M. (2012). Specific surface area and pore-size distribution in clays and shales. Geophys. Prospect..

[37] Bond F.C. (1961). Crushing and grinding calculations. Br. Chem. Eng..

[38] Gupta A., Yan D.S. (2006). Mineral Processing Design and Operations: An Introduction.

[39] King R.P. (2001). Modeling and Simulation of Mineral Processing Systems.

[40] Ford M.A., King R.P. (1984). The simulation of ore-dressing plants. Int. J. Miner. Process..

[41] Allen T. (2003). Powder Sampling and Particle Size Determination.

[42] Petrakis E., Karmali V., Bartzas G., Komnitsas K. (2019). Grinding Kinetics of Slag and Effect of Final Particle Size on the Compressive Strength of Alkali Activated Materials. Minerals.

[43] Esmaeelnejad L., Siavashi F., Seyedmohammadi J., Shabanpour M. (2016). The best mathematical models describing particle size distribution of soils. Model. Earth Syst. Environ..

[44] Coello-Velázquez A.L., Quijano Arteaga V., Menéndez-Aguado J.M., Pole F.M., Llorente L. (2019). Use of the Swebrec Function to Model Particle Size Distribution in an Industrial-Scale Ni-Co Ore Grinding Circuit. Metals.

[45] Wills B.A., Finch J. (2016). Will’s Mineral Processing Technology. An Introduction to the Practical Aspects of Ore Treatment and Mineral Recovery.

[46] Taşdemir A., Taşdemir T. (2009). A comparative study on PSD models for chromite ores comminuted by different devices. Part. Part. Syst. Charact..

[47] Magdalinović N.M. (1989). Calculation of energy required for grinding in a ball mill. Int. J. Miner. Process..

[48] Aras A., Ozkan A., Aydogan S. (2012). Correlations of bond and breakage parameters of some ores with the corresponding point load index. Part. Part. Syst. Charact..

[49] Abazarpoor A., Halali M. (2017). Investigation on the particle size and shape of iron ore pellet feed using ball mill and HPGR grinding methods. Physicochem. Probl. Miner. Process..

[50] Hlobil M., Kumpová I., Hlobilová A. (2022). Surface area and size distribution of cement particles in hydrating paste as indicators for the conceptualization of a cement paste representative volume element. Cem. Concr. Compos..

[51] Petrakis E., Komnitsas K. (2021). Development of a non-linear framework for the prediction of the particle size distribution of the grinding products. Min. Metall. Explor..

[52] Jankovic A., Sinclair S. (2006). The shape of product size distributions in stirred mills. Miner. Eng..

[53] Fuerstenau D.W., Abouzeid A.-Z.M. (1991). Effect of fine particles on the kinetics and energetics of grinding coarse particles. Int. J. Miner. Process..

[54] Cho H., Kwon J., Kim K., Mun M. (2013). Optimum choice of the make-up ball sizes for maximum throughput in tumbling ball mills. Powder Technol..

[55] Petrakis E., Komnitsas K. (2022). Effect of grinding media size on ferronickel slag ball milling efficiency and energy requirements using kinetics and attainable region approaches. Minerals.

[56] Schlepp D.D., Turner P.A. Influence of circulating load and classification efficiency on mill throughput. Proceedings of the SME Annual Meeting.

[57] Dündar H., Kalugin A., Delgado M., Palomino A., Türkistanli A., Aquino B., Lynch A.J. (2014). Screens and cyclones in closed grinding circuits. Screens and cyclones in closed grinding circuits. Proceedings of the XXVII International Mineral Processing Congress.

[58] Frausto J.J., Ballantyne G.R., Runge K., Powell M.S., Wightman E.M., Evans C.L., Gonzalez P., Gomez S. (2021). The effect of screen versus cyclone classification on the mineral liberation properties of a polymetallic ore. Miner. Eng..

